# Small Molecule APY606 Displays Extensive Antitumor Activity in Pancreatic Cancer via Impairing Ras-MAPK Signaling

**DOI:** 10.1371/journal.pone.0155874

**Published:** 2016-05-25

**Authors:** Na Guo, Zuojia Liu, Wenjing Zhao, Erkang Wang, Jin Wang

**Affiliations:** 1 State Key Laboratory of Electroanalytical Chemistry, Changchun Institute of Applied Chemistry, Chinese Academy of Sciences, Changchun, Jilin, China; 2 Department of Chemistry and Physics, State University of New York, Stony Brook, New York, United States of America; Toho University School of Medicine, JAPAN

## Abstract

Pancreatic cancer has been found with abnormal expression or mutation in Ras proteins. Oncogenic Ras activation exploits their extensive signaling reach to affect multiple cellular processes, in which the mitogen-activated protein kinase (MAPK) signaling exerts important roles in tumorigenesis. Therapies targeted Ras are thus of major benefit for pancreatic cancer. Although small molecule APY606 has been successfully picked out by virtual drug screening based on Ras target receptor, its in-depth mechanism remains to be elucidated. We herein assessed the antitumor activity of APY606 against human pancreatic cancer Capan-1 and SW1990 cell lines and explored the effect of Ras-MAPK and apoptosis-related signaling pathway on the activity of APY606. APY606 treatment resulted in a dose- and time-dependent inhibition of cancer cell viability. Additionally, APY606 exhibited strong antitumor activity, as evidenced not only by reduction in tumor cell invasion, migration and mitochondrial membrane potential but also by alteration in several apoptotic indexes. Furthermore, APY606 treatment directly inhibited Ras-GTP and the downstream activation of MAPK, which resulted in the down-regulation of anti-apoptotic protein Bcl-2, leading to the up-regulation of mitochondrial apoptosis pathway-related proteins (Bax, cytosolic Cytochrome *c* and Caspase 3) and of cyclin-dependent kinase 2 and Cyclin A, E. These data suggest that impairing Ras-MAPK signaling is a novel mechanism of action for APY606 during therapeutic intervention in pancreatic cancer.

## Introduction

Pancreatic cancer is a deadly disease due to pancreatic ductal adenocarcinoma ranking the fourth among cancer-related deaths [1]. The nature of this tumor is characterized by a poor outcome for all stages of disease and only 1–4% of pancreatic cancer patients are still alive at 5 years from diagnosis [2]. Various treatment regimens failed to significantly improve survival of patients [3,4]. Failure of chemotherapy in pancreatic cancer is mainly due to multidrug resistance and dose-limiting adverse reactions. To date, it remains unclear how intracellular signaling pathways lead to the aberrant biological properties in pancreatic cancer. Moreover, it remains little known about how pharmacological inhibitions of specific signaling pathways improve the response of pancreatic cancer cells to conventional chemotherapy [5]. Hence, future efforts toward development of novel therapy to improve survival and quality of life of patients with pancreatic cancer should include new strategy to explore effective anticancer drugs [6].

Ras proteins are key regulation components that involve in normal cell growth, differentiation and malignant transformation [7]. It was estimated that almost 90% of pancreatic cancers have been found with abnormal expression or mutation in Ras proteins [8]. Oncogenic Ras activation exploits their extensive signaling reach to affect multiple cellular processes, including suppression of apoptosis and promotion of proliferation [9]. Programmed cell death, or apoptosis, is a normal physiological process by which individual cell dies and is removed from a given population. Apoptotic cell death initiated intrinsically through the mitochondrion-mediated pathway functions as a crucial defense mechanism against malignancy, and the corruption of the apoptotic machinery is a defining signature of cancer cells [10]. Oncogenic Ras-driven erosion of the apoptotic pathway and its contribution to cancers have been well documented [11]. Among the downstream signaling cascades of Ras, the mitogen-activated protein kinase (MAPK) cascade has been reported to play important roles in the development of cancers [12–14]. One of the key roles, the Ras-MAPK pathway in a wide variety of mammalian cells, is the regulation of cell cycle transition [15]. The proliferative signals generated by oncogenic Ras culminate with the up regulation of several transcription factors triggering the expression of cyclins that attribute to the activation of the Ras-MAPK pathway. Oncogenic Ras can promote cell cycle progression by inhibiting cyclin-dependent kinases (CDKs). The suppressive effect is mediated by multiple Ras effector pathways including the Ras-MAPK pathway [16,17]. With our understanding, the contribution of oncogenic Ras to these processes will undoubtedly be an exciting avenue of cancer research in the coming future.

It is well known that small molecules have vital roles in cancer chemotherapy. A small-molecule inhibitor, APY606, was picked out by virtual drug screening based on Ras target receptor in our recent work [18]. However, its underlying mechanism of anti-cancer properties is poorly understood. Here, the in-depth investigations were performed to assess its cancer-fighting nature against pancreatic cancer Capan-1 and SW1990 cell lines. These results show that APY606-induced apoptosis is attributed to the activation of the intrinsic mitochondrial apoptotic pathway and the prevention of the Ras-MAPK pathway cascade. In parallel, APY606 was further found to induce S phase arrest and slow down the metastasis in the two cell lines by impairing Ras activation. Consequently, our research will lay the foundation for targeted Ras drug discovery and for APY606 therapeutic application in pancreatic cancer.

## Materials and Methods

### Chemicals and reagents

Dulbecco's modified Eagle's medium (DMEM), L-15 cell culture medium and fetal bovine serum (FBS) were obtained from Gibco (Grand island, NY). All the other reagents were purchased from Sigma (St. Louis, MO). APY606 was kindly provide by NCI/DTP Open Chemical Repository (http://dtp.cancer.gov) and then confirmed by HPLC and ESI-MS. Primary antibodies against human caspase-3, caspase-9, cytochrome *c*, Bcl-2, Bax, c-Raf, ERK, pERK, MEK, pMEK, cyclin A, cyclin E and CDK2 were purchased from Santa Cruz and BD Bioscience, respectively. The antibodies against human GAPDH and β-actin were obtained from Santa Cruz.

### Cell lines and cell culture

Human pancreatic cancer Capan-1 and SW1990 cell lines were obtained from the Cell Bank of Type Culture Collection Chinese Academy of Sciences (Shanghai, China) and cultured in DMEM and L-15 Medium containing 10% FBS, 100 units/mL penicillin and 100 μg/mL streptomycin, respectively. The cells were maintained at 37°C in a humidified atmosphere with 5% CO_2_ incubator. In the process of cell culture, there was not any effect of mycoplasmas on the two cell lines, which was confirmed by a fluorochrome DNA staining test. APY606 was dissolved in DMSO (dimethyl sulfoxide), and freshly diluted to the desired concentration with double distilled water immediately before use. The final concentration of DMSO in the culture media is 0.1% (v/v). The control cells received the vehicle consisting of double distilled water containing 0.1% DMSO only, which does not significantly affect the cells.

### Growth inhibition assay

The Cell Counting Kit-8 (CCK-8) (Dojindo, Japan) assay was performed to examine the effect of APY606 on cytotoxicity. Briefly, Capan-1 and SW1990 cell lines were seeded into 96-well plates at a density of 1×10^4^ cells/well in a volume of 100 μL. After overnight incubation, the cells were treated by various concentrations of APY606 for 24 h and 48 h, respectively. Cells were then exposed to 10 μL CCK-8 reagent for 3 h; the optical density at 450 nm was read with a M200 PRO NanoQuant autoreader (TECAN, Switzerland). In this assay, CCK-8 does not interfere with APY606 and cause a positive response. The measurements were carried out at least five times.

### Colony forming assay

To test the survival of cells treated with APY606, Capan-1 and SW1990 cell lines were seeded into 24-well plates (200–300 cells per well) and allowed to adhere for 24 h. The cells were incubated in culture medium containing APY606 with the concentrations of 2, 4, 6, 8 and 10 μg/mL for 6 days. After that, the cells were fixed with methanol and stained with 5% Giemsa and colonies (more than 50 cells) were counted under an inverted microscope (AMG EVOS, Life).

### Nuclear staining assay

APY606-induced nuclear condensation and morphological change were detected using DAPI (4,6-Diamidino-2-phenylindole). Capan-1 and SW1990 cell lines (5×10^6^ cell per plate) were grown on glass-bottom plates to 50% confluence and then cultured in the medium in the presence of 6.25, 12.5 and 25.0 μg/mL APY606 for 24 h, respectively. The cells were fixed with 3.5% paraformaldehyde and then incubated in a fluid containing 2 mg/mL DAPI for 20 min. The nuclear morphology of cells was observed by fluorescence microscopy (AMG EVOS, Life).

### Quantification of cell apoptosis

Early in apoptosis, phosphatidylserine (PTS) is translocated to the outer cell membrane and can be identified by binding of Annexin V, a ligand for PTS. Apoptotic Capan-1 and SW1990 cells were quantified using the Annexin V-fluorescein isothiocyanate (FITC) apoptosis detection kit (abcam, UK) after cells were treated with APY606 at different concentrations (6.25 and 12.5 μg/mL) for 24 h. Briefly, cells were trypsinized and washed twice with cold PBS, and then the cells were resuspended at a density of 1×10^6^ cells/mL in binding buffer (10 mM HEPES/NaOH, pH 7.4, 140 mM NaCl, 2.5 mM CaCl_2_). Thereafter, cells were incubated with 5 μL annexin V-FITC and 5 μL PI in the dark for 15 min at room temperature and subjected to flow cytometric analysis (FACSAria, BD Biosciences). In total, 10,000 events were analyzed in each sample. Data analysis was performed with Diva 6.0 software (BD Biosciences).

### Measurement of mitochondrial membrane potential

Disruption of mitochondrial membrane potential (ΔΨm) is a characteristic signature of apoptosis in a mitochondrial-related pathway. ΔΨm can be measured using fluorescent probe JC-1. Briefly, Capan-1 and SW1990 cell lines were seeded into 6-well plates (5×10^5^ cell per well) and treated with APY606 at concentration of 6.25 and 12.5 μg/mL for 24 h. Cells were washed with PBS and incubated in 500 μL JC-1 working solution at 37°C in 5% CO_2_ for 20 min. Thereafter, cells were resuspended in 500 μL incubation buffer, followed by visualization using confocal laser scanning microscope (CLSM, Leica, TCS Sp2). JC-1 was excited by 488 nm laser light and emission was captured at 530 nm [19]. The decrease of red/green fluorescence intensity ratio indicates the loss of ΔΨm in cancer cells.

### Determination of Ras-GTP

Capan-1 and SW1990 cell lines were cultured in complete medium for overnight, starved for 8 h in medium containing 1% FBS and then treated for 24 h with various concentrations of APY606. At the end of the treatment, cells were stimulated with EGF (10 ng) for 10 min. Thereafter, the cells were lysed and the cell lysate was processed by a Ras activation assay kit (Upstate, Millipore). The total Ras and the active Ras proteins were detected using Western blotting assay as described below.

### Flow cytometric quantification of pERK

The expression amount of phosphorylated extracellular signal-regulated kinase (pERK) was a readout of Ras-MAPK pathway cascade. To measure the inhibition extent to ERK activation experimentally, flow cytometric analysis was used to obtain quantitative single-cell measurement of the amount of pERK. Briefly, Capan-1 and SW1990 cell lines were treated with APY606 at concentration of 6.25 and 12.5 μg/mL for 24 h, then fixed and permeabilized using Cytofix/Cytoperm kit (Becton Dickinson, Mountain View). After centrifugation, 0.05 μg antibody per well in 100 μL antibody mixture for an Alexa Fluor 488 conjugated ERK1/2 antibody (anti-phospho-p44/42 MAP Kinase, Thr202/Tyr204, BD Bioscience) was added and incubated for 1 h on ice. After washing, samples were analyzed using fluorescence activated cell sorter FACSAria (BD Bioscience) and the percentage of stained cells in each quadrant was quantified using Diva 6.0 software (BD Bioscience). In total, 10,000 events were analyzed in each sample. All experiments were repeated at least three times.

### Cell cycle analysis

To test the potent mechanism for APY606-induced cell growth inhibition, the effect of APY606 treatment on cell cycle distribution was explored by flow cytometry. Briefly, Capan-1 and SW1990 cell lines were seeded (1×10^6^ cells per well in 6-well plate) and treated with APY606 at concentration of 6.25 and 12.5 μg/mL for 24 h, respectively. Cells were suspended, fixed in 70% (v/v) ethanol at 4°C overnight. Thereafter, cells were washed and resuspended in 1 mL PBS containing 50 μg/mL PI and 1 mg/mL RNaseA at room temperature in dark for 30 min. In total, 10,000 events were analyzed immediately in each sample by flow cytometer (FACSCalibur, BD Biosciences). All experiments were repeated at least three times.

### Wound healing assay

To explore the influence of APY606 on the motility ability of cancer cells, wound healing assay was conducted to evaluate the cell motility of APY606-treated cells. Capan-1 and SW1990 cell lines were plated onto 24-well culture dishes and then scored using a micropipette tip. The medium was replaced by 12.5 μg/mL APY606 in the complete medium, and the cell migration was monitored using CLSM for 24 h. Images were captured, and the wound closure distance (compared to control at 0 h) was measured in three-independent wound sites per group. Relative cell motility is compared as the wound width difference between at 0 h and at 24 h.

### Trans-well chamber assay

To further examine the effect of APY606 on the invasion ability of cancer cells, Matrigel trans-well chamber assay was carried out. Capan-1 and SW1990 cell lines were seeded onto 6-well plates at a density of 2×10^5^ cells/well and cultured for 24 h. After cells were starved for 24 h, cells were treated with 12.5 μg/mL APY606. Thereafter, cells were collected and 1×10^5^ cells diluted with serum free medium were plated to trans-well units with polycarbonate filters (Cambridge, MA) containing 8-μm pores. The polycarbonate membrane was pre-coated with 250 μg/mL Matrigel (BD Biosciences). The bottom chambers were filled with 600 μL medium containing 5% FBS. After 24 h, the cells were fixed in methanol and stained with Acridine Orange (AO, Dingguo, China). The top surface of the membrane was gently scrubbed with a cotton bud, and the cells invading through the filters membrane were counted on glass bottom culture plates, and images corresponding to the entire membrane surface were captured by CLSM.

### Western blotting analysis

In order to clarify the underlying mechanisms for APY606-induced cell apoptosis and cell cycle arrest at molecular level, Western blotting analyses were examined. Capan-1 and SW1990 cell lines were washed with cold PBS after 24 h treatment with APY606 at concentration of 6.25 and 12.5 μg/mL. After cellular proteins were extracted and quantified, equal amount of protein was electrophoresed on 10% SDS-PAGE gel and then transferred onto polyvinylidene difluoride (PVDF) membrane (Millipore, Bedford, MA). Thereafter, PVDF membrane was probed with the indicated primary antibody overnight at 4°C and further blotted with appropriate horseradish peroxidase-conjugated secondary antibody. Visualization was performed using chemiluminescence detector (DNR, Kiryat Anavim, Israel). Protein level was normalized using GAPDH or β-actin as an internal control.

### Statistical analysis

Data are presented as mean ± SD of triplicate experiments. Statistical analysis was conducted using SPSS 11.5 statistical software.

## Results

### 1. APY606 inhibits proliferation of Capan-1 and SW1990 cell lines

**Fig 1:**
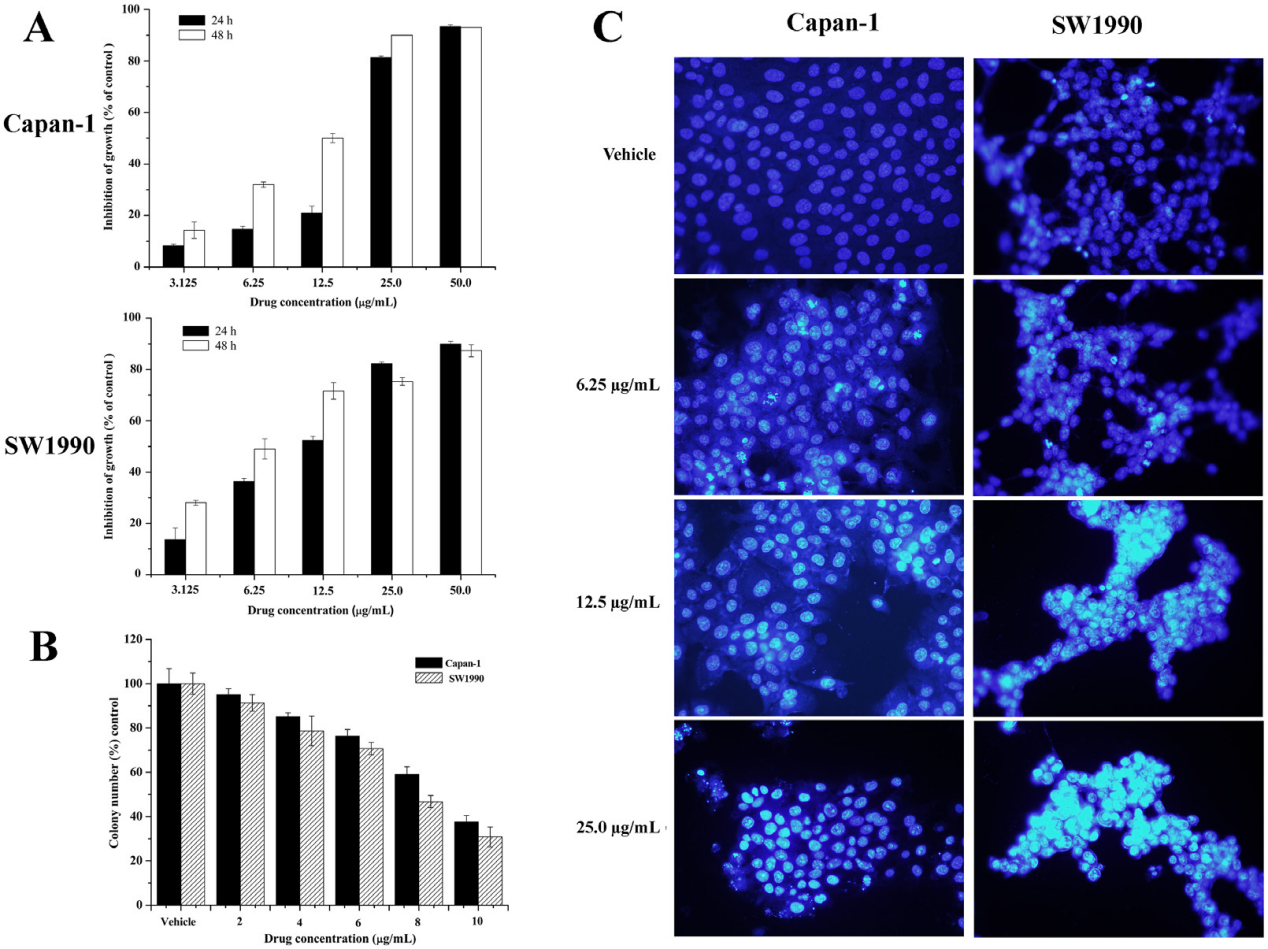
APY606 inhibits the proliferation and growth of Capan-1 and SW1990 cell lines. Cells were treated with indicated concentrations of APY606 for 24 and 48 h and then assessed by CCK-8 (A) and colony forming (B) assay, respectively. Presented data are the mean±SD of three independent experiments. Nuclear staining images (C) were taken by fluorescent microscope with 10× objective. The scale bar was 20 μm.

To explore the potential growth inhibition of APY606 in the two cancer cell lines, the concentration-dependent inhibitory effects of APY606 on the growth of Capan-1 and SW1990 cell lines were evaluated as shown in Fig 1A. When Capan-1 cells were treated with APY606 at concentration of 12.5, 25.0 and 50.0 μg/mL for 24 h, the percentage of growth inhibition over the control cells (100%) was nearly 21.0±2.6%, 81.3±0.5% and 93.3±0.6%, respectively. Accordingly, the percentage of inhibited SW1990 cells over the control cells (100%) was 52.3±1.5%, 82.3±0.6% and 90.0±1.0%, respectively. When Capan-1 cells were treated with APY606 at the same conditions for 48 h, the percentage of growth inhibition over the control cells (100%) was approximately 50.0±1.7%, 90.0% and 93.0%, respectively. Meanwhile, the percentage of inhibited SW1990 cells over the control cells (100%) was 71.7±3.2%, 75.3±1.5% and 87.3±2.3%, respectively. The IC_50_ value for APY606 was 14.3±1.3 μg/mL (24 h) and 9.5±0.6 μg/mL (48 h) in Capan-1 cells as well as 10.3±1.1 μg/mL (24 h) and 6.8±2.3 μg/mL (48 h) in SW1990 cells, respectively. In addition, we have also studied the effect of APY606 on the survival rate of Capan-1 and SW1990 cell lines by colony forming assay. APY606 treatment resulted in a significant inhibition of colony formation in the two cell lines in a dose-dependent manner (Fig 1B). Collectively, our results indicated that APY606 induced concentration-dependent inhibitory effects on the growth and survival of both Capan-1 and SW1990 cell lines.

Further, the nuclear morphology of cells was detected by DAPI that is a blue fluorescence dye specially binding A-T rich regions in DNA. DAPI can pass through an intact cell membrane therefore it can be used to stain both live and fixed cells, though it passes through the membrane less efficiently in live cells and therefore the effectiveness of the stain is lower. The blue fluorescence intensity in Capan-1 and SW1990 cell lines treated with APY606 at concentration of 6.25, 12.5, and 25.0 μg/mL for 24 h was stronger than that in control groups treated with vehicle as shown in Fig 1C. Moreover, it is clear that the condensed and fragmented nuclei increased with the APY606 treatment in a concentration-dependent manner.

### 2. APY606 induces apoptosis of Capan-1 and SW1990 cell lines

**Fig 2:**
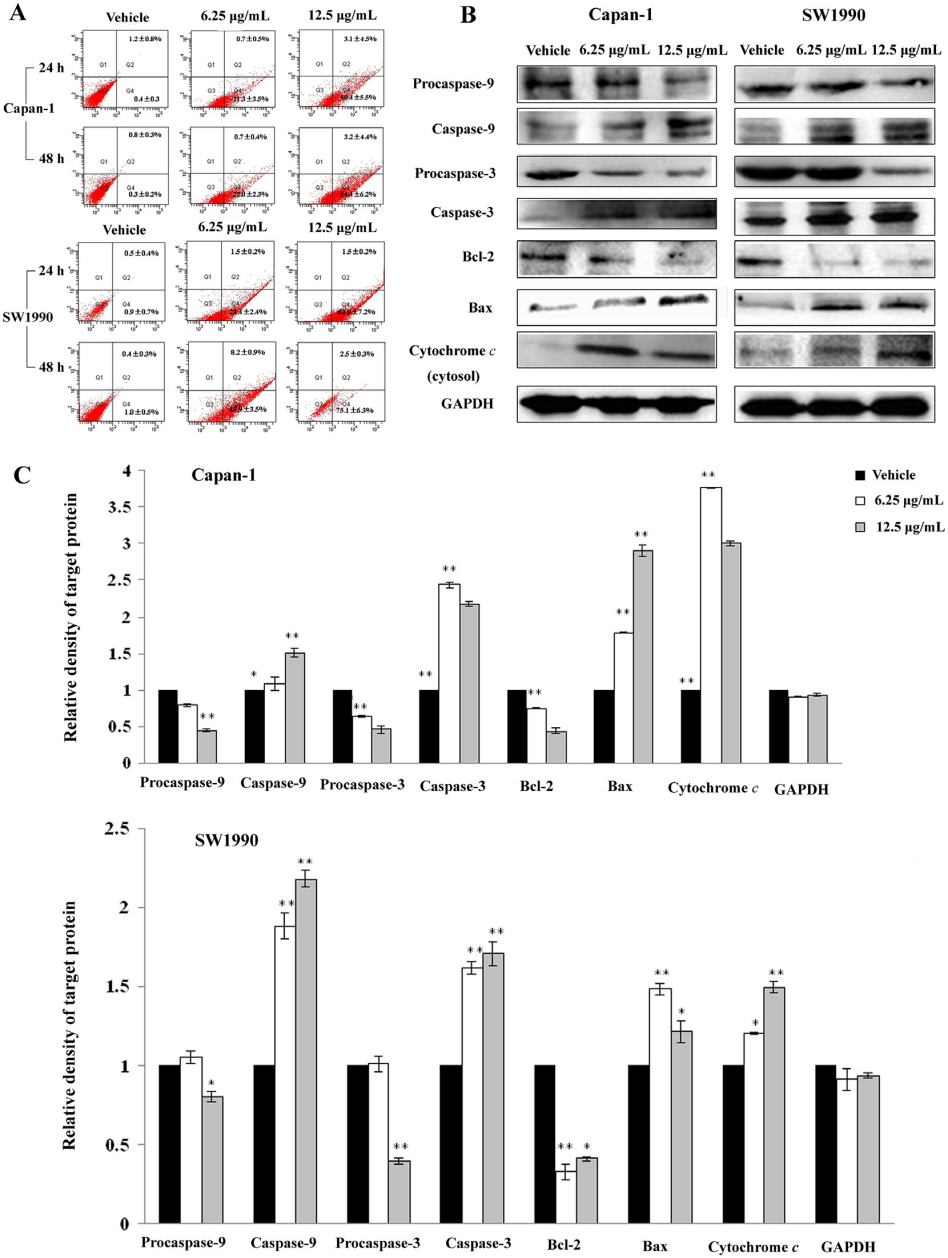
APY606 induces apoptosis in Capan-1 and SW1990 cell lines. Percentages of apoptotic cell populations in the two cell lines treated with 6.25 and 12.5 μg/mL of APY606 for 24 and 48 h were determined by using flow cytometer (A). Presented data are the mean±SD of three independent experiments. Effect of APY606 treatment on the proteins related to apoptotic pathway was measured using Western blotting analysis. Representative blots of respective proteins were displayed (B). GAPDH was used as the internal control. (C): Relative density of target protein was quantitated and plotted.

To evaluate the apoptosis-inducing effect of APY606 on Capan-1 and SW1990 cell lines, the number of apoptotic cells was quantified using flow cytometric analysis. Cells stained positive for annexin V-FITC and negative for PI are undergoing apoptosis; cells stained positive for both annexin V-FITC and PI are either in the end stage of apoptosis undergoing necrosis or already dead; cells stained negative for both annexin V-FITC and PI are alive without undergoing apoptosis [20]. As shown in Fig 2A, the number of apoptotic cells was negligible (0.4–1.0%) in the control cells of the two cell lines treated with vehicle. While the percentage of early apoptotic cells was increased to 21.3±3.5% and 50.4±5.5% after Capan-1 cells were treated with APY606 at 6.25 and 12.5 μg/mL for 24 h, respectively. For 48 h treatment, the values were increased to 22.0±2.3% and 54.4±6.2%, respectively. Similarly, at the same conditions, the values were 23.4±2.4% and 63.9±7.2% as well as 49.9±3.5% and 75.1±6.3% after SW1990 cells were exposed to APY606 for 24 h and 48 h, respectively. The results clearly showed that APY606 induced a time- and concentration-dependent apoptosis in Capan-1 and SW1990 cell lines.

To investigate the mechanism responsible for APY606-induced apoptosis, we evaluated the levels of Bax, Bcl-2, cytosolic cytochrome *c*, and the activation of caspase-3 and caspase-9 in Capan-1 and SW1990 cell lines treated with APY606 at concentration of 6.25 and 12.5 μg/mL for 24 h using Western blotting analysis. The effects of APY606 treatment on the expression levels of the pro-apoptotic protein Bax and anti-apoptotic protein Bcl-2 were experimentally examined. Treatment of cells significantly increased the expression level of Bax but decreased that of Bcl-2 in a concentration-dependent manner (Fig 2B). Cytochrome *c* is one of the central mediators of the mitochondrial or intrinsic apoptotic pathway. The release of cytochrome *c* from the mitochondrial intermembrane space is the early event during apoptotic cell death [21]. As such, the effects of APY606 treatment on the release of cytochrome *c* in the two cell lines were evaluated. Exposure of cells to APY606 was observed to increase the release of cytochrome *c* from mitochondria into cytosol in a concentration-dependent manner (Fig 2C). Upon apoptotic stimulation, cytochrome *c* released associates with procaspase-9 to form a complex processing caspase-9 from inactive proenzyme to its active form, eventually triggering caspase-3 activation and apoptosis [21]. As shown in Fig 2B and 2C, APY606 induced remarkably concentration-dependent activation of caspase-3 and caspase-9 and eventually led to apoptotic death in Capan-1 and SW1990 cell lines.

### 3. APY606 induces ΔΨm

The depletion of ΔΨm is an early and essentially apoptotic response to anti-cancer therapy. Mitochondrial disruption initiates the process of apoptosis, which subsequently leads to growth inhibition. To further explore the mechanism for APY606-induced cell apoptosis, we determined the effect of APY606 on ΔΨm in Capan-1 and SW1990 cell lines. The cationic dye JC-1 is useful to detect ΔΨm occurring at the early stage of apoptosis [22]. In living cells, JC-1 exhibits potential dependent accumulation in mitochondria leading to the concentration-dependent formation of red fluorescence J-aggregates [23] that is indicative of the presence of polarized mitochondria. On depolarization, JC-1 monomer binding with mitochondrial membrane results in green fluorescence and a reduction in orange-red staining. Thus, mitochondrial depolarization is indicated by a decrease in the red/green fluorescence intensity ratio. The ratio of red to green fluorescence is dependent only on the membrane potential and not on other factors such as mitochondrial size, shape and density that may influence single-component fluorescence signals.

**Fig 3:**
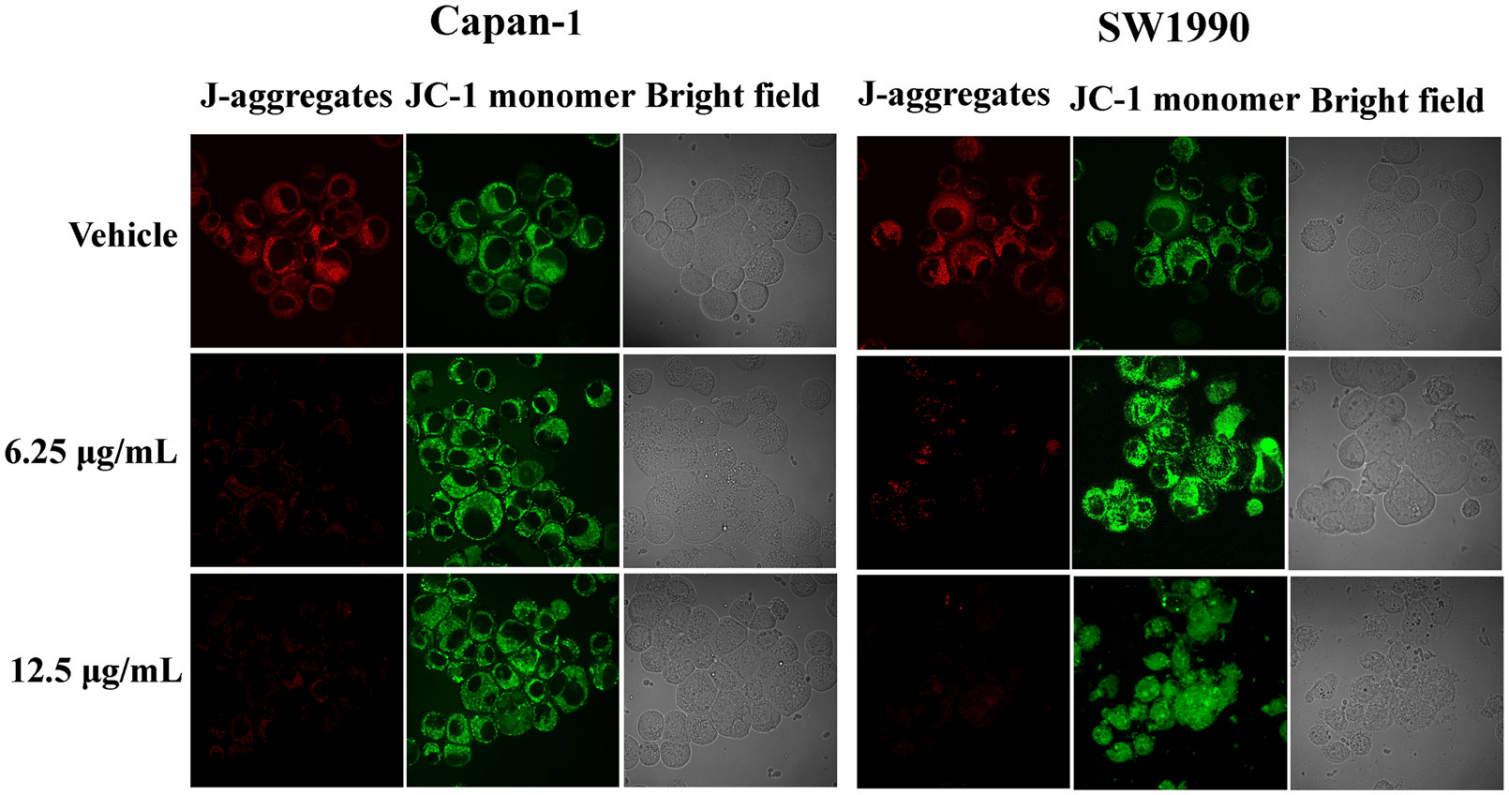
Mitochondrial membrane depolarization was examined using fluorescent probe JC-1 in Capan-1 and SW1990 cell lines. The control and APY606-treated cells stained with JC-1 were monitored by CLSM. The J-aggregate form (red fluorescence) and J-monomer alone (green fluorescence) were excited at 568 and 480 nm, respectively. The scale bar was 20 μm.

Here, the control and APY606-treated Capan-1 and SW1990 cell lines stained with JC-1 were monitored by CLSM. As shown in Fig 3, the formation of red-fluorescent J-aggregates was significantly reduced in the two cell lines treated with 6.25 and 12.5 μg/mL APY606 compared to the control cells. In contrast, the green fluorescence was particularly noted in APY606-treated cells owing to JC-1 monomer binding. The ratio of green to red fluorescence was increased in APY606-treated cells in a concentration-dependent manner, suggesting that the fluorescence change was indicative of ΔΨm. Loss of ΔΨm was observed under APY606 treatment, suggesting that mitochondria are affected particularly early during the apoptotic process.

### 4. APY606 inhibits Ras-MAPK pathway inducing apoptotic response

**Fig 4:**
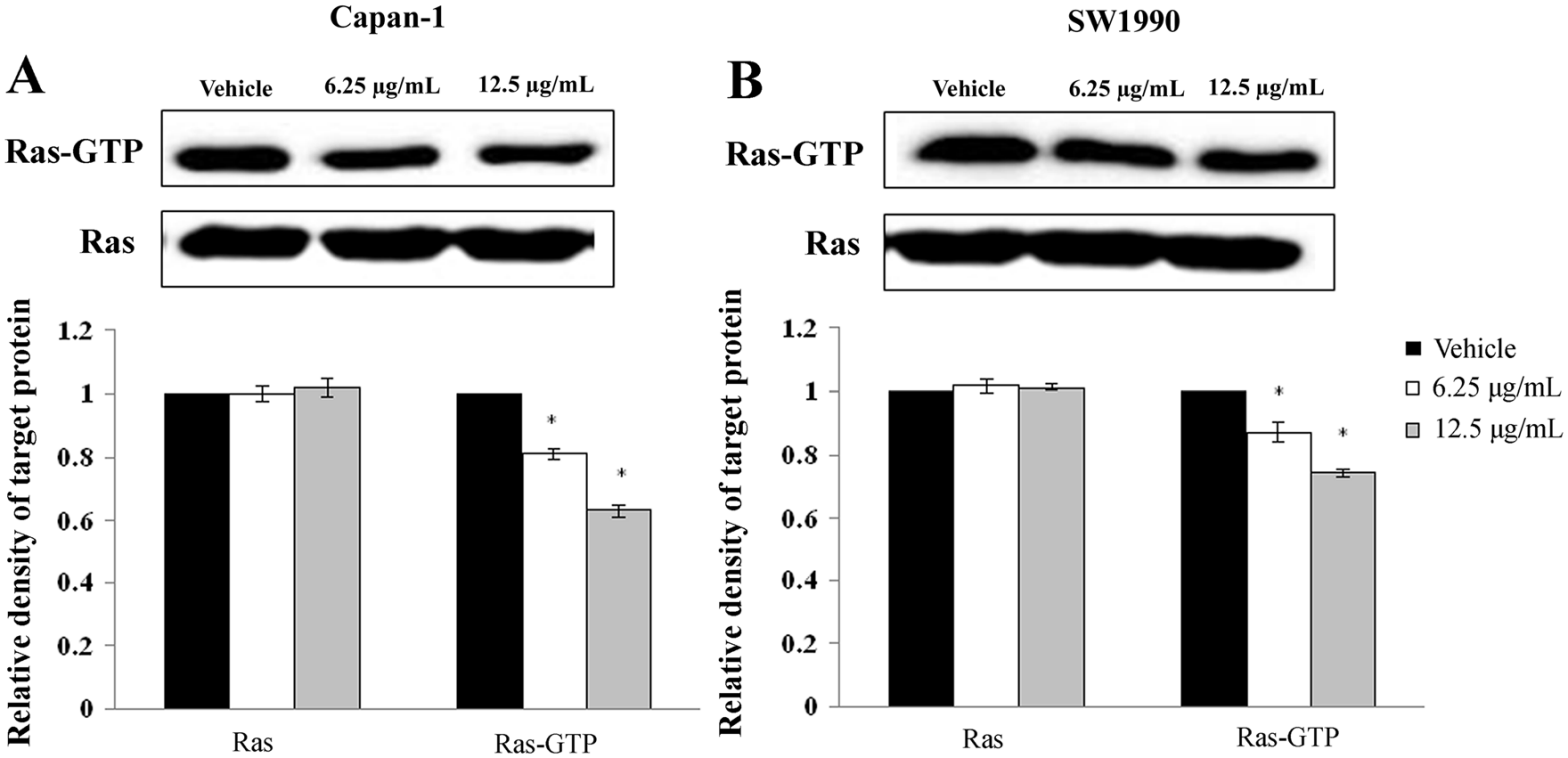
APY606 attenuates Ras activation. Serum-starved cells were treated with vehicle or indicated concentrations of APY606 for 24 h, and then stimulated with EGF for 10 min. APY606 attenuates cellular Ras activation in Capan-1(A) and SW1990 (B) cell lines. GTP-bound Ras was isolated by RBD pull-down assay and detected by Ras-GTP activation kit. Total amount of Ras was detected by anti-Ras specific antibody. Relative density of target protein was quantitated and plotted.

Aiming to assess the possibly targeted Ras therapeutic strategy, we first examined the inhibition of Ras-GTP activity in cell-based assay. In theory, APY606 will act to diminish the Ras-GTP activity level. To account for this prediction, the effect of APY606 on pancreatic cancer cells was evaluated using the Raf1RBD pull-down assay. As predicted, the extents of Ras activation in serum-starved Capan-1 and SW1990 were substantially reduced in the presence of APY606 in a dose-dependent manner without a significant decrease in total Ras level (Fig 4).

**Fig 5:**
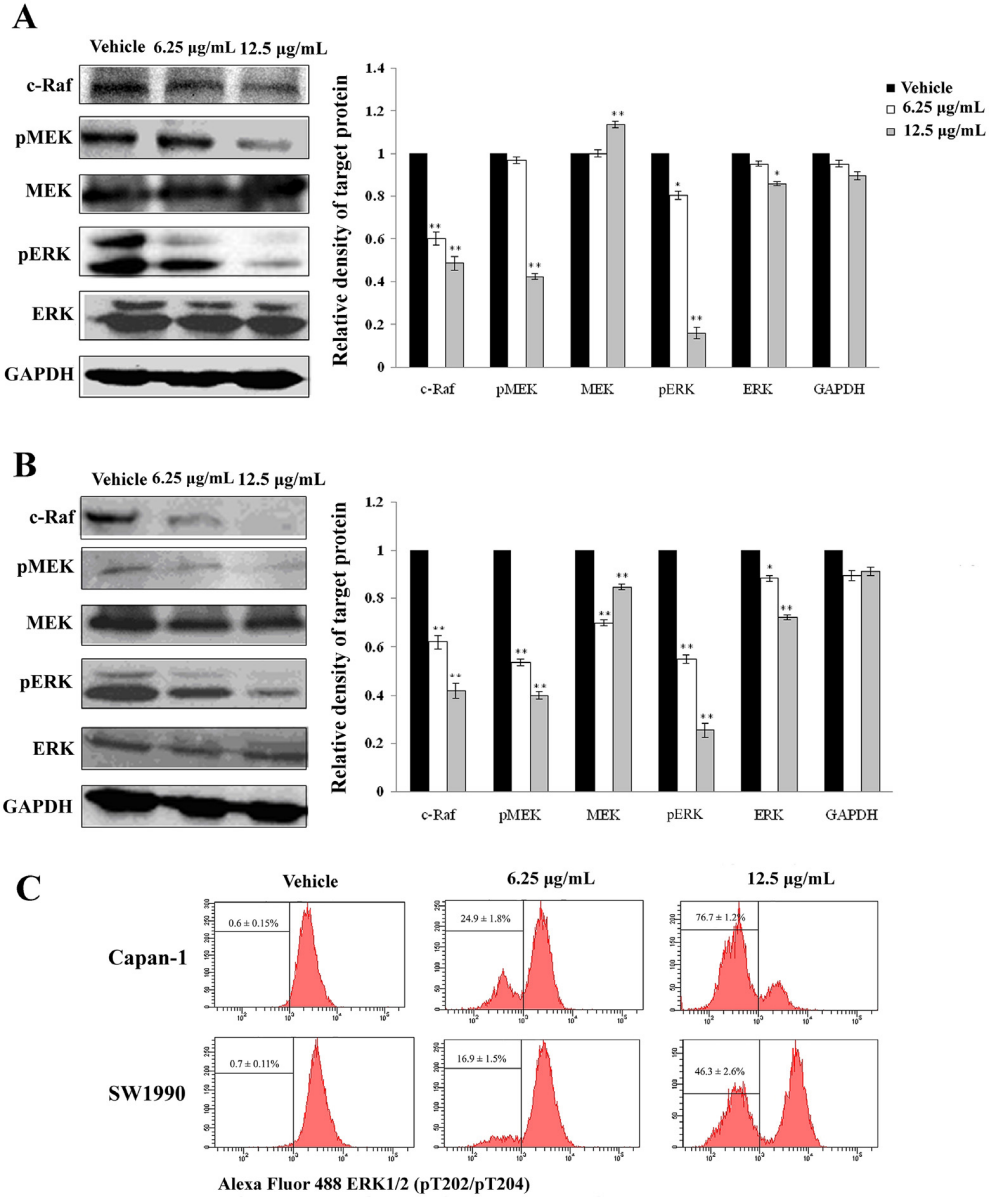
Effect of APY606 on inhibition of Ras-MAPK signaling pathway in Capan-1 (A) and SW1990 (B) cell lines. Cellular lysates were analyzed in immunoblotting with respective primary antibody followed by the second antibody and the representative blots were measured. GAPDH was used as the internal control. Relative density of target protein was quantitated and plotted. Flow cytometric detection of phospho-ERK was performed in both Capan-1 and SW1990 cell lines (C). Presented data are the mean±SD of three independent experiments.

Raf kinases are the best known as key regulators of the Ras-MAPK cascade, and the block of Ras-MAPK signaling pathway has an important role in induction of cancer cell apoptosis [24]. To check the underlying mechanism of apoptosis induction, we evaluated the effect of APY606 on Ras-MAPK pathway in both Capan-1 and SW1990 cell lines. Treatment of the two cell lines with APY606 at 6.25 and 12.5 μg/mL for 24 h significantly decreased the phosphorylation of MEK and ERK compared to the control cells (Fig 5A and 5B). However, exposure of the two cell lines to APY606 did not affect the total levels of MEK and ERK. Notably, the expression level of c-Raf was significantly decreased with increasing concentration of APY606 when compared to the control cells. This result showed that APY606 induced apoptosis by blocking the Ras-MAPK signaling pathway.

In general, the extent of pERK was regarded as an indicator of Ras activation [25]. Based on this, we simultaneously performed a flow cytometric analysis for obtaining quantitative single-cell measurement. As shown in Fig 5C, APY606 caused production of less pERK in Capan-1 (76.7±1.2%) and SW1990 (46.3±2.6%) cell lines at 12.5 μg/mL dose than did in vehicle-treated control cells, respectively, in agreement with the tendency of Western blotting analysis.

### 5. APY606 arrests the cell cycle in S phase

**Fig 6:**
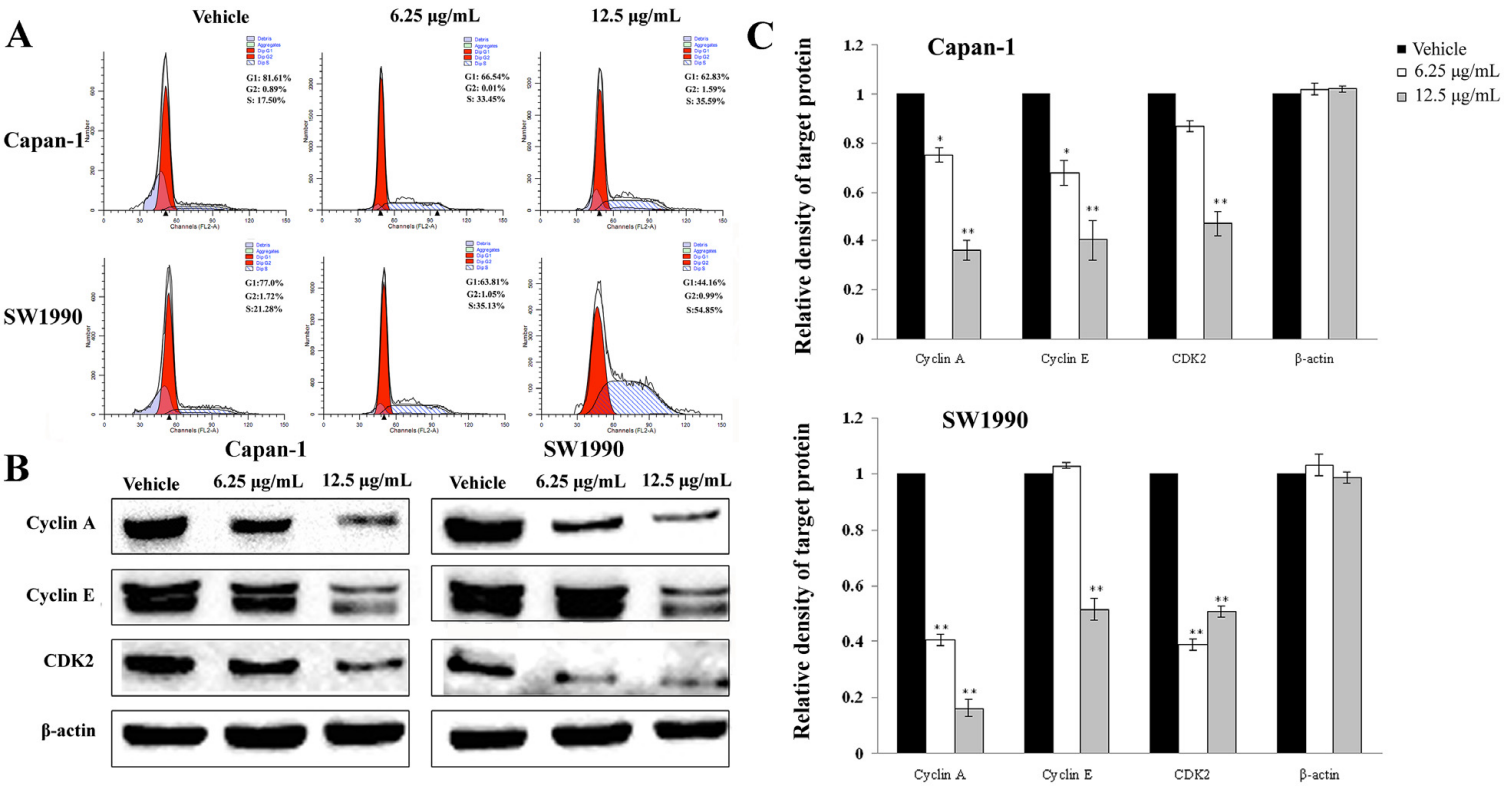
Cell cycle analysis of APY606-treated cells. Capan-1 and SW1990 cell lines were treated with indicated concentrations of APY606 for 24 h. Representative percentages of cell populations in G1, S, and G2 phases of the cell cycle in the two cell lines were analyzed by flow cytometry (A). G1-S transition-related proteins were analyzed using Western blotting assay (B). Similar experiments were repeated at least three times with similar results, and the representative blots were presented. β-actin was used as the internal control. Relative density of target protein was quantitated and plotted (C).

Many cytotoxic agents arrest cell cycle at G1, S or G2-M phase [26]. To assess the mechanism for APY606-induced cell growth inhibition, the effect of APY606 on cell cycle distribution was first explored quantitatively in Capan-1 and SW1990 cell lines using flow cytometric analysis. Treatment of the two cell lines with APY606 at 6.25 and 12.5 μg/mL for 24 h markedly did affect the number of cells in G1 and S phases at a concentration-dependent manner, but the number of cells in G2 phase was not signally changed compared to the control cells (Fig 6A). After treatment of Capan-1 cells with APY606 at 12.5 μg/mL, the number of cells arrested in G1 phase was 62.83% compared to control cells; this resulted in a decrease by 18.78% (p = 0.027). Simultaneously, the percentage of cells arrested in S phase was 35.59%; this gave an increase by 18.09% (p = 0.043) compared to the control cells treated with vehicle only. For the distribution of SW1990 cells in G1 and S phases, exposure of cells to APY606 gave rise to a more remarkable effect. Treatment of SW1990 cells with APY606 at 12.5 μg/mL significantly reduced the number of cells arrested in G1 phase by 32.84% (p = 0.002) and increased the number of cells in S phase by 33.57% (p = 0.042), respectively. These data indicated that APY606 resulted in growth inhibition that elicited a prominent, prolonged accumulation of cells in S phase and a reduction of cells in G1 phase in both Capan-1 and SW1990 cell lines.

Cell cycle progression is tightly regulated by cyclins and CDKs. The reduced expressions of cyclin A, cyclin E and CDK2 are the hallmarks of cell cycle arrest in S phase. In order to qualitatively examine the mechanism for APY606-induced cell cycle arrest, we discussed the effect of APY606 treatment on the expression levels of cyclin A, cyclin E and CDK2 in Capan-1 and SW1990 cell lines using Western blotting assay. Treatment of cells with APY606 at 6.25 and 12.5 μg/mL for 24 h significantly suppressed the expression levels of cyclin A, cyclin E and CDK2, respectively, compared to the control cells (Fig 6B). Taken together, our study demonstrated that APY606 induced remarkable cell cycle arrest in S phase in both Capan-1 and SW1990 cell lines. The cell cycle arrest could be partially ascribed to down-regulation of cyclin A, cyclin E and CDK2 expression (Fig 6B and 6C).

### 6. APY606 suppresses the metastasis and invasion of cancer cells

**Fig 7:**
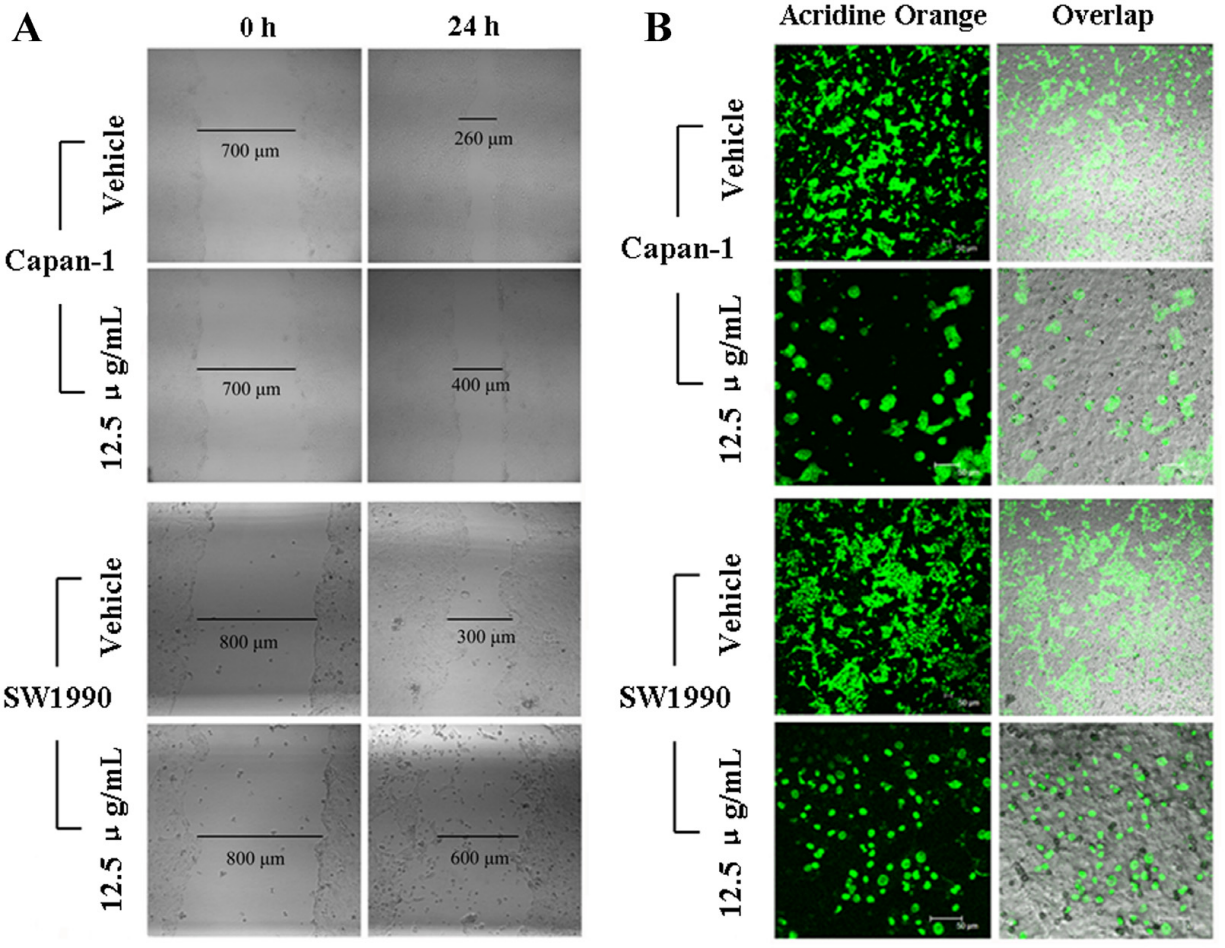
APY606 suppresses the metastasis and invasion of Capan-1 and SW1990 cell lines. The wound closure was assessed at 24 h after cells being treated with vehicle or 12.5 μg/mL APY606 (A). The distance of wound closure (compared with control at 0 h) was measured in three-independent wound sites per group. The invasion assay was carried out using Matrigel-coated trans-well culture chambers and invading cells stained with AO were photographed using CLSM (B). The images of cells on the bottom side of the filters membrane were taken in three sites per group. The scale bar was 20 μm.

Increased cell metastasis and invasion of cancer cells are key steps in the metastatic cascade [27]. Cell migration and invasion are very important components in the spread of pancreatic cancer. We therefore examined the effect of APY606 on the motility and invasion ability of both Capan-1 and SW1990 cell lines. Wound healing assay was conducted to evaluate the cell motility after cells were exposed to APY606. The wound closure was assessed at 24 h after the two cell lines being treated with vehicle or 12.5 μg/mL APY606. As shown in Fig 7A, APY606 reduced the ability to close the wound by approximate 1.5-fold and 2.5-fold for Capan-1 and SW1990 cell lines, respectively. The invasion assay was carried out using Matrigel-coated trans-well culture chambers and invading cells were counted using image analysis. As shown in Fig 7B, APY606 intuitively suppressed the invasion for Capan-1 and SW1990 cells. The results collectively indicate that APY606 has ability to suppress the metastasis and invasion of pancreatic cancer.

## Discussion

**Fig 8:**
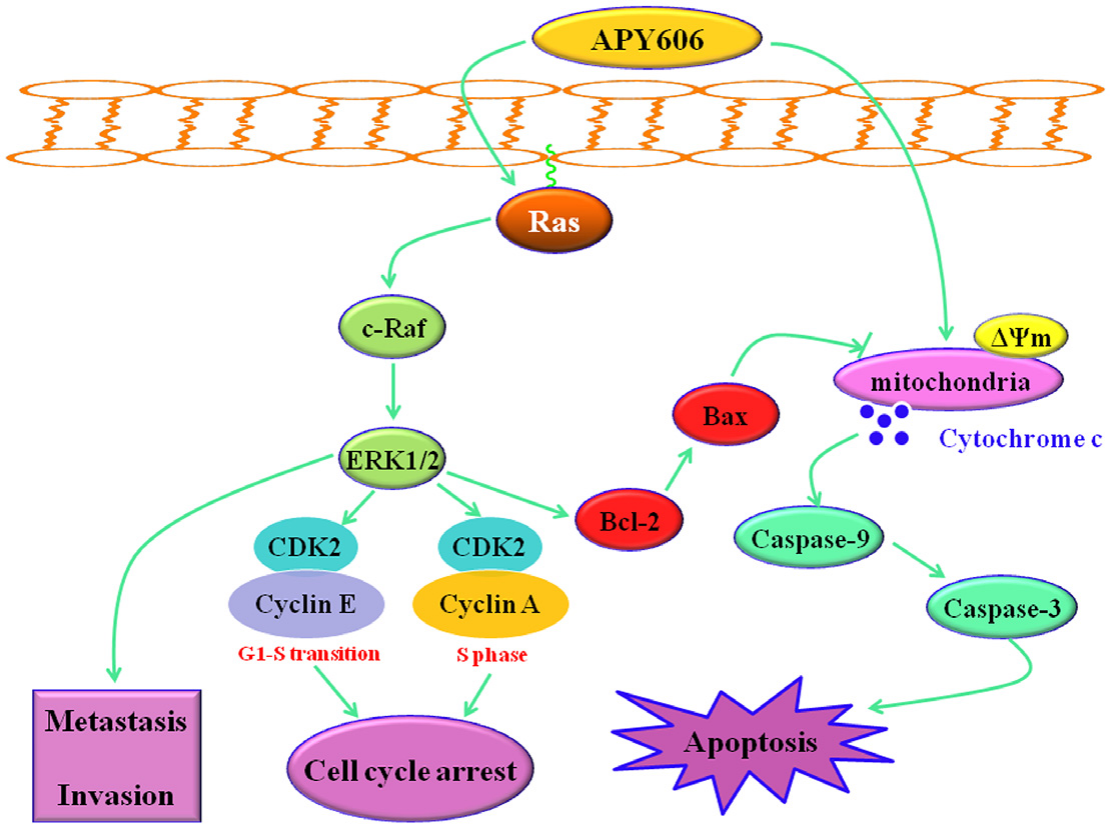
Pathway overview of APY606-induced apoptosis, cell cycle arrest, and metastasis and invasion inhibition.

Cancer is described as a disease that involves excessive proliferation of cells and abandonment of their ability to die [28]. Normally, cells can kill themselves in a balanced process known as programmed cell death or apoptosis, which represents an exquisitely efficient cellular suicide pathway. It is becoming clear that little cell suicide by apoptotic process can lead to a variety of cancers, and then cancer cells evade apoptosis to support malignant growth [29]. In cancer, the therapeutic goal is to trigger tumor-selective cell death. The induction of apoptosis in cancer cells has been recognized as an innovative drug discovery strategy for cancer therapy [27]. Therefore, understanding the mechanism of apoptosis and designing therapeutic approach to trigger cell death in cancer cells are critical for effectively treating cancers. The treatment of pancreatic cancer remains a major challenge owing to poor efficacy and severe toxicity of standard and new chemotherapies. There is an increased interest in seeking new therapy for pancreatic cancer from inducing apoptosis agents [27]. In the present study, we observed a potent inhibitory effect of APY606 on the growth of both Capan-1 and SW1990 cell lines. We found APY606 promoted cell apoptosis, arrested the cell cycle in S phase, and suppressed the metastasis and invasion in the two cancer cell lines through inhibition of Ras-MAPK signaling pathway and activation of the mitochondria-mediated apoptotic pathway (Fig 8).

The present study has shown that APY606 displayed the remarkable concentration-dependent inhibitions of proliferation and colony formation on Capan-1 and SW1990 cell lines (Fig 1). Such an inhibitory effect can be attributed to the pro-apoptotic effect of APY606. The mechanism by which APY606 exerts its apoptotic effect on cancer cells remains an interesting topic to be elucidated. In the process of apoptotic cell death, two central pathways are known: one of them is initiated by ligation of the death receptors at the cell surface in the so-called extrinsic pathway; another is induced by DNA damage or endoplasmic reticulum stress leading to apoptosis by the release of cytochrome *c* from mitochondria, known as intrinsic pathway [30,31]. The proteolytic activity of caspase-3 and caspase-9 is marker for the mitochondrial pathway. Caspase-9 can be activated by the release of apoptosis promoting factors involved in a mitochondrial pathway, e.g. cytochrome *c*, from mitochondria to the cytosol leading to recruitment and activation of caspase-3. The effects of APY606 on apoptosis were examined quantitatively and qualitatively in our study (Fig 2). After being treated by APY606, the cleavages of caspase-3 and caspase-9 were increased in a dose-dependent manner. In addition, the expression of Bax was up-regulated concomitant with the related attenuation of Bcl-2 protein expression. Mitochondria commit to apoptosis via the decreased ΔΨm and the release of cytochrome *c*. Following the treatment of Capan-1 and SW1990 cell lines with APY606, we observed that APY606 induced cytochrome *c* release from the mitochondria into the cytosol. Moreover, disruption of the ΔΨm is considered to be one of the apoptotic process induced by chemotherapeutic drugs [32]. To further assess the effects of APY606 on the mitochondrial apoptotic pathway, the ΔΨm was measured using fluorochrome dye JC-1. As shown in Fig 3, APY606 treatment resulted in a concentration-dependent intuitive decrease in the red fluorescence intensity and increase in the green fluorescence intensity, respectively. These observations reveal that APY606-induced apoptosis was partly regulated by the intrinsic mitochondrial apoptotic pathway in Capan-1 and SW1990 cell lines.

Mechanistically, the Ras-MAPK pathway can downregulate pro-apoptotic mediator such as Bax or upregulate anti-apoptotic molecule such as Bcl-2. Thus, ERK activation can contribute to Ras-induced suppression of apoptosis. Although the context in which the pro-apoptotic or anti-apoptotic capability of Ras is enacted remains unclear, it is the balance of pro-survival and pro-apoptotic signals that ultimately determine whether cells will shift towards life or death [10]. Considerable evidence indicates that Ras-MAPK signaling cascade regulates not only cell growth, development and differentiation, but also apoptosis and cell cycle arrest [30,33]. To understand the mechanism by which APY606 affects Ras-MAPK signaling pathway, the role of APY606 in the activation of Ras-MAPK pathway was determined. Western blotting assay showed that the Ras-GTP activity and phosphorylation of MEK and ERK was gradually decreased after APY606 treatment, but the total level of MEK and ERK was hardly affected (Figs 4, 5A and 5B). At the flow cytometric single cell level, the tendency was consistent with the protein expression level (Fig 5C). From the results obtained so far, it can be concluded that the cell apoptosis of Capan-1 and SW1990 cell lines induced by APY606 was mediated by prevention of the Ras-MAPK signaling pathway.

The cell cycle is a series of events taking place in a cell and essential for understanding cell growth, proliferation, development and death [34]. To further examine how endogenous Ras function was related to the cell cycle transition machinery, we investigated whether APY606 could interfere with the cell cycle as a leading phenomenon of the observed apoptosis in both Capan-1 and SW1990 cell lines. Apoptosis may occur at any stages of cell cycle, however, the transition from G1 to S phase is regarded as a crucial point for deciding between cell growth and apoptosis [35]. We found that APY606 resulted in a progressive increase in the population of cells in S phase in a concentration-dependent manner (Fig 6A). For example, 24 h after treatment, compare to the control cells, the S phase population accounted was increased by approximate 36% and 55% for Capan-1 and SW1990 cell lines treated with 12.5 μg/mL APY606, respectively. In contrast, compare to the control group, the G1 phase population were reduced from 81.61% to 62.83% for Capan-1 and 65.59% to 44.61% for SW1990 cells treated with 12.5 μg/mL APY606 by affecting the transition from G1 to S phase.

Cell cycle is a process involving many regulations among genes. Recently, our global sensitivity analysis based on landscape and flux has explicitly pointed out that cell cycle dynamics is controlled by the underlying gene regulatory networks formed by cyclins and CDKs, which together drive the cell-cycle progression [34,36]. Progression from G1 to S phase is regulated by a number of the cyclin family members, in particular cyclin A and cyclin E. Meanwhile, CDK2 is pivotal for the G1-S phase transition and mitosis of the cancer cell cycle. From our prediction of cell-cycle networks mode, it clearly shows that the strength of cyclin A, cyclin E and CDK2 activation significantly promotes the cell cycle. When cyclin A, cyclin E and CDK2 activation decrease, which means that the force from the flux is smaller than the backward force from the potential gradient, suggesting that the barrier_G1/S_ becomes larger and then the cell cycle stops at G1-S phase. The cell cycle arrest means the cell cycle period is significantly increased. Experimentally, we therefore explored the effect of APY606 on cyclin A, cyclin E and CDK2 in the two cell lines. These results (Fig 6B and 6C) suggest that inhibition of proliferation of both Capan-1 and SW1990 cell lines by APY606 involves S phase arrest, mainly via inhibiting cyclin A, cyclin E and CDK2 activation, in agreement with our prediction.

Pioneer studies showed that the Ras-MAPK pathway was well described as mediator of Ras, which was required for tumor cell transformation and invasion. During the development of pancreatic cancer, primary cancer cells will move out and invade adjacent issues or circulate to distant organs to form new clones, which is responsible for about 90% death of the affected patients. Thus, new chemotherapeutic agents that can inhibit the metastasis of pancreatic cancer cells will be available for prolonging the survival time of the patients. As shown in Fig 7, APY606 slowed down the mobility and suppressed the invasion of Capan-1 and SW1990 cell lines. Taken together, APY606 showed excellent bioactivity of controlling metastasis and invasion in pancreatic cancer cells.

## Conclusions

In conclusion, the data presented in this study reveal that APY606 can indeed promote apoptosis in pancreatic cancer cells through activating the intrinsic mitochondrial apoptotic pathway and blocking the Ras-MAPK signaling pathway, arrest cell cycle transition in S phase and suppress the metastasis and invasion in Capan-1 and SW1990 cells. This will provide a new strategy for the development of clinical application for pancreatic cancer treatment. Therefore, our study suggests that APY606 may be a novel chemotherapeutic agent against pancreatic cancer.
